# Widespread application of apomixis in agriculture requires further study of natural apomicts

**DOI:** 10.1016/j.isci.2024.110720

**Published:** 2024-08-14

**Authors:** Charity Z. Goeckeritz, Xixi Zheng, Alex Harkess, Thomas Dresselhaus

**Affiliations:** 1 HudsonAlpha Institute for Biotechnology, Huntsville, AL 35806, USA; 2 Cell Biology and Plant Biochemistry, University of Regensburg, 93040 Regensburg, Germany

**Keywords:** Agricultural science, Applied sciences, Biological sciences, Biotechnology, Molecular biology, Natural sciences, Plant biology

## Abstract

Apomixis, or asexual reproduction through seeds, is frequent in nature but does not exist in any major crop species, yet the phenomenon has captivated researchers for decades given its potential for clonal seed production and plant breeding. A discussion on whether this field will benefit from the continued study of natural apomicts is warranted given the recent outstanding progress in engineering apomixis. Here, we summarize what is known about its genetic control and the status of applying synthetic apomixis in agriculture. We argue there is still much to be learned from natural apomicts, and learning from them is necessary to improve on current progress and guarantee the effective application of apomixis beyond the few genera it has shown promise in so far. Specifically, we stress the value of studying the repeated evolution of natural apomicts in a phylogenetic and comparative -omics context. Finally, we identify outstanding questions in the field and discuss how technological advancements can be used to help close these knowledge gaps. In particular, genomic resources are lacking for apomicts, and this must be remedied for widespread use of apomixis in agriculture.

## Graphical abstract

**Figure:**
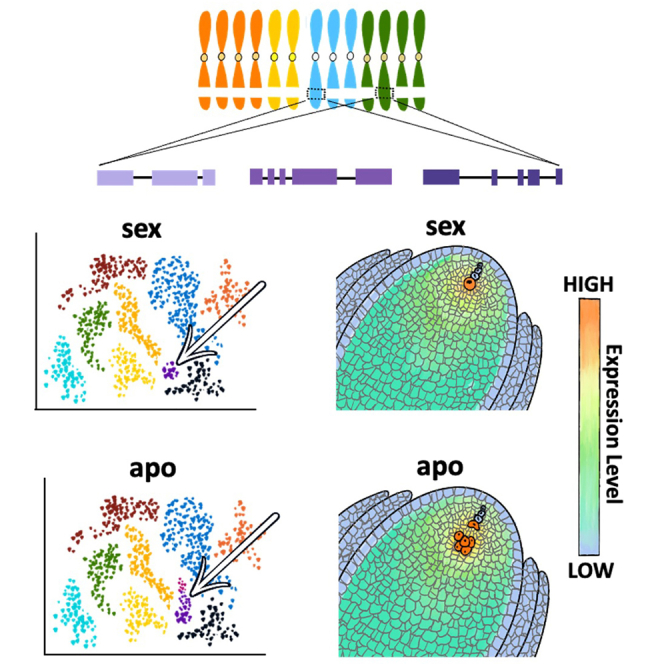

## Introduction

Apomixis is defined as asexual reproduction through seeds and results in progenies that are genetically identical to the mother plant.^1^ Its regular application in diverse crops would revolutionize agriculture as clonal F1 hybrid seeds with fixed heterosis can be indefinitely preserved and generated at low cost.^2^ It results in the immediate fixation of any desired genotype, thus allowing further investment in more diverse germplasm and greatly shortening breeding times.^3^ Apomictic reproduction also has the potential to increase seed set in genotypes that would otherwise be expected to be infertile (e.g., triploid and higher-ploidy hybrids), as evident by the main mode of reproduction of such individuals in some natural populations.^4^ For these reasons, there is interest in dissecting the molecular mechanisms underlying apomixis for incorporation into breeding schemes. Many excellent reviews written in the last several years have discussed the challenges pertaining to this goal and summarized current findings at the genetic level mostly in a few apomictic model species.^5^^,^^6^^,^^7^^,^^8^

**Figure 1:**
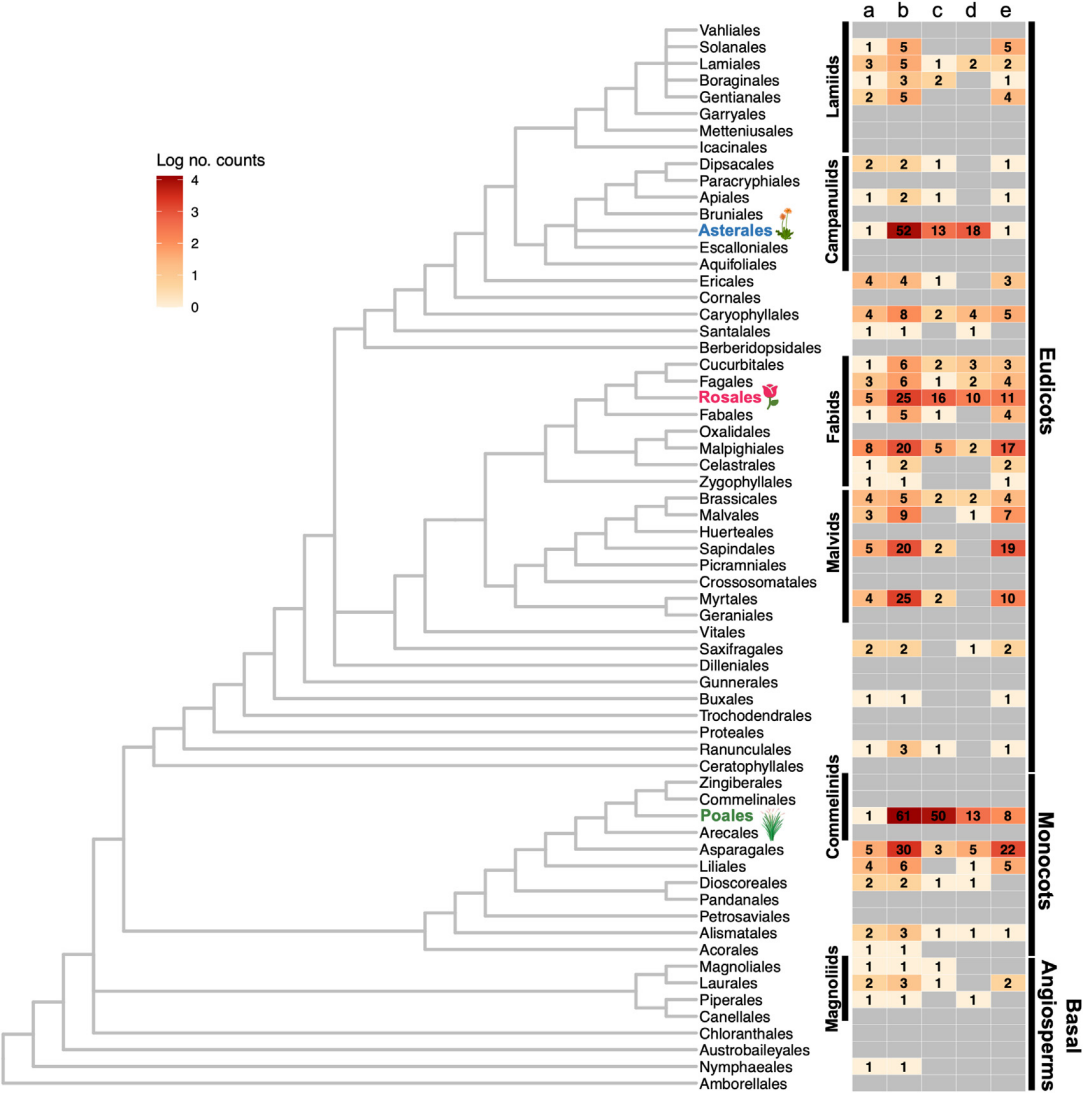
Apomixis is a convergent trait with documented cases scattered throughout the flowering plant phylogeny Phylogenetic relationships of flowering plant orders according to the Angiosperm Phylogeny Group classification IV are shown alongside a heatmap with 5 columns. (a) Indicates the number of families in the order with documented cases of apomixis; (b) indicates the number of genera, followed by the number of cases of documented (c) apospory, (d) diplospory, and (e) adventitious embryony. The color of each cell is proportional to the log number of counts. Gray cells are instances where no known cases have been documented thus far. Data were taken from the apomixis database created by Hojsgaard et al.^9^ and were recounted in September 2023. The Asterales, Rosales, and Poles are highlighted on the phylogeny as they contain most known gametophytic apomicts. Certain clades as well as eudicots, monocots, and basal angiosperms are also indicated.

In contrast, the present perspective focuses on the importance of studying natural apomicts in diverse flowering plants using emerging technologies. Decades of research have led to the discovery of several apomixis genes in a small handful of model taxa; however, apomixis has independently evolved more than one hundred times in more than half of the flowering plant orders. In angiosperms, it has been documented in 34 orders, 80 families, and 326 genera^9^ (Figure 1) and is especially frequent in the Asterales, Rosales, and Poales. Moving forward, studying the repeated origins of apomixis across the phylogeny and within diversity collections is a powerful approach to complement the discovery of novel pathways since various genes have been shown to control the trait in different lineages.^10^^,^^11^^,^^12^ Gene discovery in non-model apomictic plants using a wide phylogenetic framework doubly ensures the successful application of apomixis. First, it complements synthetic approaches through discovery and functional characterization of novel apomixis genes. Second, by examining apomixis in related species to crops, it reduces the chances of pleiotropic effects caused by wide evolutionary distances, thereby increasing the feasibility of introgressing the trait into genotypes that may not be amenable to transformation.

## The genetic control of apomixis

The convergent nature of apomixis requires a brief review on the types and mechanisms of this complex trait. Apomixis is generally divided into two major types depending on the origin of the embryo: sporophytic and gametophytic apomixis. These two types of apomixis have independently evolved throughout the angiosperm phylogeny, with examples of families exhibiting sporophytic apomixis including Orchidaceae and Rutaceae and examples of families exhibiting gametophytic apomixis including Asteraceae, Rosaceae, and Poaceae (Figure 1).

In sporophytic apomixis (also known as adventitious embryony), unreduced embryos originate directly from somatic cells of the ovule. An embryo produced by sporophytic apomixis matures alongside the fertilized sexual embryo and competes for resources from the developing endosperm, which presents a challenge in achieving fully penetrant clonal seeds and thus diminishes its appeal in plant breeding.^8^ Sporophytic apomixis is frequent in Malpighiales, Sapindales, and Asparagales. Gametophytic apomixis consists of several components: (1) apomeiosis, in which an ovule cell bypasses meiosis and recombination to produce an unreduced embryo sac, (2) parthenogenesis, or embryo development without fertilization, and (3) endosperm formation, whether that be automatically (autogamy) or triggered by fertilization of the central cell (pseudogamy). Gametophytic apomixis is further broken down into apospory and diplospory, depending on the origin of the unreduced embryo sac. In aposporic species, the unreduced embryo sac emerges from a somatic cell of the ovule that assumes megagametophyte-like properties and may coexist with the reduced (sexual) embryo sac depending on the environment and genotype; ultimately these factors seem to govern which mode of reproduction prevails.^13^^,^^14^^,^^15^^,^^16^^,^^17^^,^^18^ Diplospory is considered a deregulation of the sexual process since the origin of unreduced female gametophytes is the megaspore mother cell (MMC).^19^

Apomixis has a complex evolutionary pattern, which is reflected in the genetic architecture of the trait. It should be stated that apomeiosis and parthenogenesis have historically been treated as qualitative traits, even though research clearly indicates variation in penetrance due to genetic background.^20^ Genetic mapping studies have shown different genes separately controlling each of the three components, and genetic loci regulating the components of apomixis have been found to be linked and more often inherited together (Poaceae,^11^^,^^21^^,^^22^ Hypericaceae^23^), or unlinked, exhibiting a 1:1 segregation pattern in subsequent generations (Asteraceae, Rosaceae^13^^,^^23^^,^^24^^,^^25^). These loci may be in hemizygous regions surrounded by repetitive sequences, so recombination may be suppressed (but not always, e.g., *Taraxacum*) and large mapping populations may be necessary to identify them since recombination between tightly linked loci is rare.^21^^,^^24^ While these rare events have been instrumental to our current understanding of the genetic architecture of apomixis, genetic mapping methods are laborious and time-consuming—not to mention extraordinarily complicated for polyploids, a notable characteristic of most apomicts.^9^ Consequently, the master determinants for parthenogenesis, a component of apomixis, have only been definitively validated in two apomictic species, and “apomeiosis” and “autonomous endosperm development” genes have yet to be discovered in natural apomicts. For a more thorough discussion of candidate genetic determinants relevant to apomixis, readers are encouraged to review Table 1 and references therein from Xu et al.^8^ In the next sections, we briefly highlight what is known for the genetic control of apomixis components and how these findings have been used to improve synthetic apomixis, all while making the case for further study of natural apomicts.

### Apomeiosis-related genes

Cloning the causal genes in apomicts has been a historically challenging endeavor due to limited genomic resources, frequent occurrences of polyploidy, and low recombination between genetic loci. Mainly, associations have been made between loci or candidate genes and apomeiosis.^8^ For example, a candidate identified in *Poa pratensis*, called APOSTART_6 (a total of 15 APOSTART cDNAs have been identified), co-segregates with apomixis and shows specific expression in floral tissues.^26^ Similarly, a long non-coding RNA theorized to regulate expression of *QUI-GON JINN*, a gene that appears to affect aposporous embryo sac formation, co-segregates with apospory in *Paspalum notatum.*^27^ The *DIPLOSPOROUS* (*DIP*) locus associates with unreduced female gamete formation in *Taraxacum*,^25^^,^^28^ and the *LOSS OF APOMEIOSIS* (*LOA*) locus regulates apospory in *Pilosella piloselloides* (formerly *Hieracium praealtum).*^24^ Strong but correlative evidence attributed certain *APOLLO* alleles with apomicts in a diverse collection of *Boechera* accessions,^29^^,^^30^ and recent experiments indicated the 5′ UTR of the APOLLO apomictic allele is important for expression in reproductive tissues in *Arabidopsis.*^31^ Still, it remains to be seen if these regulatory features and/or the APOLLO protein sequence are necessary and sufficient to induce apomeiosis in either *Boechera* or *Arabidopsis*. Arguably the most promising evidence for candidate apomeiosis genes was recently demonstrated through the characterization of *Arabidopsis TRIMETHYLGUANOSINE SYNTHASE1* (*TGS1*). A *TGS1* homolog was first identified as a candidate for apospory in apomictic *Pasplaum notatum*, and the null allele of the *Arabidopsis* homolog results in the emergence of an extra cell exhibiting developmental properties similar to the MMC.^32^

### Induction of parthenogenesis

In contrast to apomeiosis, identification of genes governing the second component of gametophytic apomixis—parthenogenesis—has been met with tremendous success in recent years. The first breakthrough in decades emerged through investigation of the natural apomict *Pennisetum squamulatum* (Poaceae), when several *BABY BOOM-LIKE* (*BBML*) AP2 transcription factors were discovered in the apospory-specific genomic region (ASGR).^11^ *PsASGR-BBML* transgenes were able to induce parthenogenesis in monocots like pearl millet,^11^ rice,^33^ and maize,^34^ and there is also evidence to suggest conservation of *BBML* genes in apospory-associated loci for other Panicoideae grasses, like *Cenchrus ciliaris* (buffel grass) and *Brachiaria humidicola* (Koronivia grass).^35^^,^^36^ For eudicots, *PsASGR-BBML* failed to induce parthenogenesis in *Arabidopsis*^34^ but could trigger parthenogenesis in tobacco at a low frequency (1%–9%), depending on the egg cell-specific promoter used to drive its expression.^37^

Another major step toward understanding parthenogenesis in natural apomicts was achieved when a gene was identified and cloned from *Taraxacum officinale.*^10^ *PARTHENOGENESIS* (*PAR*) encodes a putative transcriptional repressor containing a K2-2 zinc finger and an EAR (ethylene-responsive element binding factor-associated amphiphilic repression) domain. A MITE (miniature inverted-repeat transposable element) insertion in the *ToPAR* promoter is essential for its expression in the apomictic dandelion egg cell. Notably, a MITE was also detected in the promoter of *PAR* genes in apomictic *Pilosella piloselloides*, suggesting parallel evolution of apomixis driven by MITE insertion in Asteraceae.^10^ Interestingly, several *MITE* insertions in the promoter of a *RWP-RK* gene are thought to induce nucellar embryogenesis (sporophytic apomixis) in *Citrus* and *Fortunella*.^38^ Taken together, the identification of *PsASGR*-*BBML* and *ToPAR* confirms the multiple origins of (gametophytic) apomixis since nature has commandeered different genes in different lineages for asexual reproduction.

### Endosperm development in apomicts

Endosperm is the major storage organ for nourishing the developing embryo or seedling; without it, the seed will abort.^39^ Most apomictic species are pseudogamous, meaning endosperm formation requires fertilization of the central cell,^40^ and only a few apomictic Asteraceae species are known to spontaneously form endosperm (autonomous endosperm) without fertilization. In the case of autonomous endosperm formation, the maternal genome is in excess relative to the typical 2:1 maternal:paternal endosperm ratio required for most sexual species.^41^ Some pseudogamous apomicts are also able to tolerate deviations from this ratio,^42^^,^^43^^,^^44^^,^^45^ and understanding these relaxed endosperm constraints will be important for interploidy crosses and for introgressing apomictic traits into crops. However, identifying genes for endosperm formation has been largely unsuccessful. One study showed a negative correlation between expression levels of a *FERTILIZATION INDEPENDENT ENDOSPERM* (*FIE*) homolog with apomictic seed formation in *Malus hupehensis*,^46^ and a more recent one implicated an isogene of *ORIGIN OF RECOGNITION COMPLEX 3* (*ORC3*) in *Paspalum* apomicts in relaxing the endosperm balance ratio requirement.^45^ A dominant genetic locus for autonomous endosperm formation was mapped in *Hieracium* and *Taraxacum*, but the variable penetrance of the trait indicates additional genetic factors are likely involved.^16^^,^^47^

## Advances in synthetic apomixis

### *Mitosis instead of meiosis* combined with haploid induction methods

**Figure 2:**
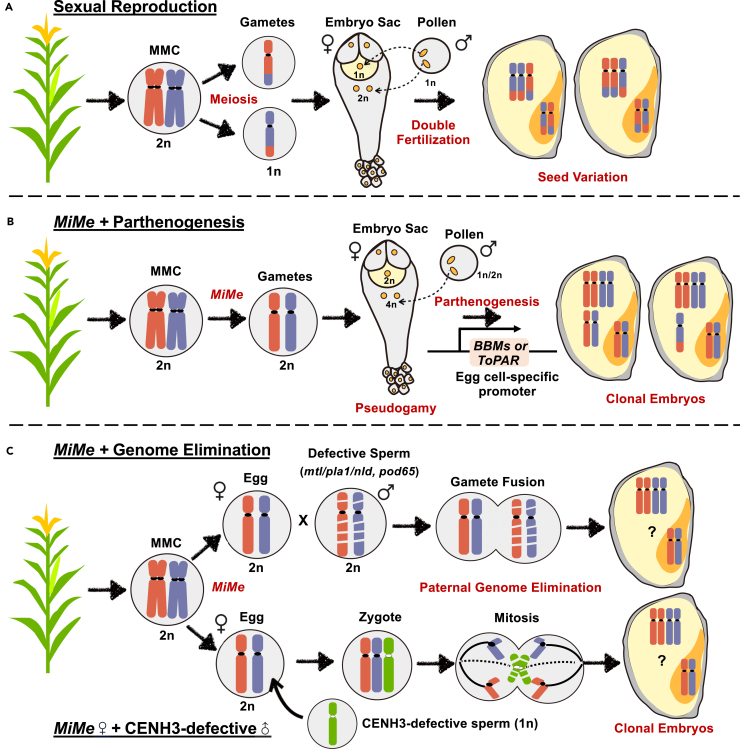
Overview about strategies to engineer synthetic apomixis in crops (A) During sexual reproduction of crop plants like maize, the diploid (2n) megaspore mother cell (MMC) undergoes meiosis producing reduced and recombined haploid (1n) gametes. After double fertilization, the resulting seeds in the next generation will exhibit variation. (B) The combination of the *MiMe* triple mutant system with ectopic expression of *BBMs* or *ToPAR* in the egg cell enables the generation of clonal embryos and seeds. Unreduced egg cells develop into diploid clonal embryos via parthenogenesis, while the 4n central cell can be fertilized with unreduced 2n or haploid 1n sperm cells (pseudogamy), respectively. (C) An alternative strategy to generate clonal embryos is to combine the *MiMe* system with defective sperm cells leading to uni-parental (male) genome elimination. Defective sperm cells carry either *mtl/pla/nld*, *dmp*, *pod65*, or *pld3* mutants (above scenario) or a CENH3-defective mutant (bottom scenario) leading to male chromosome segregation defects and ultimately their elimination. The chromosome composition of the endosperm is unclear (question marks).

During sexual reproduction, the diploid (2n) MMC undergoes meiosis resulting in haploid gametes (1n) that contain reduced and recombined chromosomes (Figure 2A). After double fertilization, embryos will exhibit significant variation and are genetically distinct from the mother plant. A major goal in plant breeding and biotechnology is to circumvent meiosis to engineer synthetic apomixis. This trait became a feasible option for asexual and clonal seed production after years of investigating meiotic mutants in the sexual plant *Arabidopsis.*^48^^,^^49^^,^^50^^,^^51^^,^^52^ Mutations in at least three genes (e.g., *spo11-1/rec8/osd1*) define the genetic background of *Mitosis instead of Meiosis* (*MiMe*), which essentially phenocopies apomeiosis.^48^ However, due to double fertilization, *MiMe* alone leads to the doubling of ploidy levels in successive generations and must be coupled with either haploid induction techniques or parthenogenesis for true asexual seed formation. Several haploid induction techniques have been developed in combination with *MiMe* for double haploid creation and synthetic apomixis (Figure 2B) and will be introduced in the following paragraphs.

### *Mitosis instead of meiosis* with parthenogenesis genes discovered in natural apomicts

The combination of parthenogenesis genes identified in natural apomicts and the *MiMe* system has shown to be very successful in generating clonal seeds. Haploid induction rates depend on the *BBM* homolog used, the method to create egg cell-specific expression, the species, and the genotype. Ectopic expression of *BABYBOOM* homologs (*OsBBMs*) in egg cells of rice led to haploid induction rates between 3% (*At*p*DD45*:*OsBBM4*) and 29% (*At*p*DD45*:*OsBBM1*).^53^^,^^54^ CRISPR-dCas9-mediated *ZmBBM2* egg cell-specific activation led to ∼2% haploid induction in maize,^55^ and ectopic expression of *ZmBBM1* driven by the egg cell-specific promoter *AtEC1.2* achieved efficiencies of up to 74%.^56^ ToPAR, the parthenogenesis gene isolated from dandelion, has also been used to induce haploids in *Setaria italica* (foxtail millet) at a rate of up to 10.2%.^57^

Aside from *Arabidopsis*, *MiMe* has been applied to rice^58^^,^^59^ and tomato^60^ with different intended outcomes for each crop. In tomato, researchers used *MiMe* to create tetraploid tomatoes with enhanced heterosis, demonstrating the wide applicability of synthetic apomixis outside of clonal seed production.^60^ In rice, *MiMe* was implemented with the intention of obtaining fully clonal seed, and, until recently, it was met with limited success. The latest advancements include a single CRISPR-Cas9 cassette containing multiple guide RNAs to create *MiMe* and egg cell-specific expression of genes *OsBBM* and *ToPAR,* which has led to high rates of clonal seed production in rice. *sgMiMe_pAtECS:BBM1* and *sgMiMe_pOsECS:BBM1* plants show clonal seed rates up to 95%; however, these plants show a 16% reduction in fertility compared to the wild type.^33^ Conversely, *sgMiMe_pDD45:BBM4* plants have a low clonal seed production rate of 1%–2% but fertility is largely unaffected.^53^ Similar genetic constructs using the PAR gene isolated from dandelion (sgMiMe_pAtEC1.1:ToPAR) resulted in the production of 40%–60% clonal seeds with no significant impacts on fecundity.^61^ These frequencies were mostly stable in respective generations, but improvements are needed to combat deleterious effects on fertility.^33^ In the future, the co-expression of different parthenogenesis-related genes may result in better penetrance of clonal seed production without fertility defects.

### Other haploid induction methods combined with *MiMe*

Haploid induction can also occur via genome elimination of one of the parental genomes (Figure 2C). One method includes the use of CENH3 mutants, deficient in functional centromeric histone H3 protein (CENH3), which guides the assembly of kinetochores and chromosome segregation.^62^ CENH3 modification to induce haploids has been applied in maize,^63^ wheat,^64^ and other crops.^65^ However, combining *MiMe* with CENH3 genome elimination has only been achieved in *Arabidopsis*, and only 34% of the seeds were clonal after the first generation and 24% in the second.^66^ This method also relies on the availability of sexually compatible *cenh3* mutants for crossing with genotypes intended for asexual propagation; thus, testing its potential for clonal seed generation is currently limited. Still, it could become a viable option for engineering synthetic apomixis in the future.

Other possibilities for haploid induction involve specific genetic factors of the pollen parent. One of the most impactful includes a phospholipase A1 called *ZmMTL/ZmPLA1/NLD*, the gene underlying the quantitative trait locus (QTL), *qhir1*, in maize haploid inducer line Stock 6.^67^^,^^68^^,^^69^ Further study of the mutant implicated oxidative stress in a mechanism that leads to paternal genome fragmentation before gamete fusion.^70^ These researchers also identified *ZmPOD65*, which encodes a sperm-specific peroxidase that modulates haploid induction. Soon thereafter, mutants of another pollen-specific phospholipase, *ZmPLD3*, were shown to triple the haploid induction rate when combined with null *ZmMTL/ZmPLA1/NLD* alleles.^71^

Another strategy for haploid induction might include a membrane protein first characterized in Stock 6 called *ZmDMP.*^72^ Moreover, compared with the *mtl* single mutant, double mutants for *mtl dmp* increase the haploid induction rates from ∼1% to 7%.^72^ *DMP* orthologs are also present in dicots, and there are reports for loss-function *DMP*-like genes inducing haploids in *Arabidopsis*,^73^ tomato,^74^ *Brassica napus*, and tobacco,^75^ meaning these genes may have broader potential for plant breeding in the future. As for the mechanism, *AtDMP8* and *AtDMP9* were shown to participate in the process of double fertilization. In the *dmp8 dmp9* double mutant, the fusion of mutant sperm cells with egg cells is especially defective, often resulting in a single, preferential fertilization event of the central cell.^76^ Similarly, *ECS1* and *ECS2* encode egg cell-specific endopeptidases that also regulate the double fertilization process and could be used in haploid induction strategies. Double mutants *ecs1 ecs2* show unsuccessful fusion of sperm cell and egg nuclei after fertilization, thereby leading to maternal haploids.^77^^,^^78^ Finally, mutants of another gene regulating the double fertilization process, AtKPL, cause maternal haploid induction.^79^

To our knowledge, only *mtl* has been used in combination with *MiMe* in a crop; unfortunately, it had variable success in rice as only 9 of 145 progeny were true maternal clones and the seed-setting rate was reduced to 6%.^59^ With more research, it is hoped that mutations in the genes described earlier could be used in combination for high haploid induction rates and asexual seed production.

### Necessity of autonomous endosperm development

It is debatable whether it is necessary to engineer autonomous endosperm formation, since in agricultural settings pollen availability is usually not a limiting factor for production. However, this trait is attractive for plant breeding for two reasons. First, pollen exclusion in autonomous apomicts guarantees that the asexually produced egg cell will not be fertilized. Second, it facilitates the adoption of pollen-sterile plants to prevent pollen transfer and undesirable introgression into sexual crop fields. On this front, research in rice demonstrated *Osfie1* and *Osfie2* double mutants exhibit a high frequency of asexual embryo and autonomous endosperm formation,^80^ but embryo abnormality and lethality as well as incomplete stages of endosperm development must be understood and remedied before the application of these genes in synthetic apomixis.

## A case for studying apomixis in its phylogenetic context

As outlined earlier, it has become clear in the past decade that haploid induction through genome elimination and other methods has had limited success for usage in engineering apomixis and that the greatest gains were achieved using the synthetic *MiMe* system combined with parthenogenesis genes discovered in natural apomicts. Therefore, it is in our best interest to continue identifying loci governing the components of apomixis in natural apomicts to complement synthetic approaches and guarantee broader use of apomixis in agriculture.

These observations warrant important considerations as researchers attempt *MiMe* in more crops. It is largely unknown how the expansion and contraction of these gene families will affect their predicted functions across lineages. Outcomes of gene duplication (e.g., through whole-genome duplication) include neo- and subfunctionalization.^81^ Thus, across larger evolutionary distances, it is reasonably likely that the homologs of these genes identified in *Arabidopsis* confer new or only partial functions in other lineages, which may introduce pleiotropic defects— including loss of fertility. Duplications also introduce the technical challenge of knocking out additional homologs of *MiMe*, while the identification of genes administering apomeiosis (which are known to have dominant effects) in different lineages of natural apomicts offers the potential of more reliably engineering the trait with a single gene. Therefore, targeted study of natural apomicts within a phylogenetic clade that includes a major crop should be top priority.

### Third-generation sequencing technologies and comparative genomics for the identification of apomictic loci

**Figure 3:**
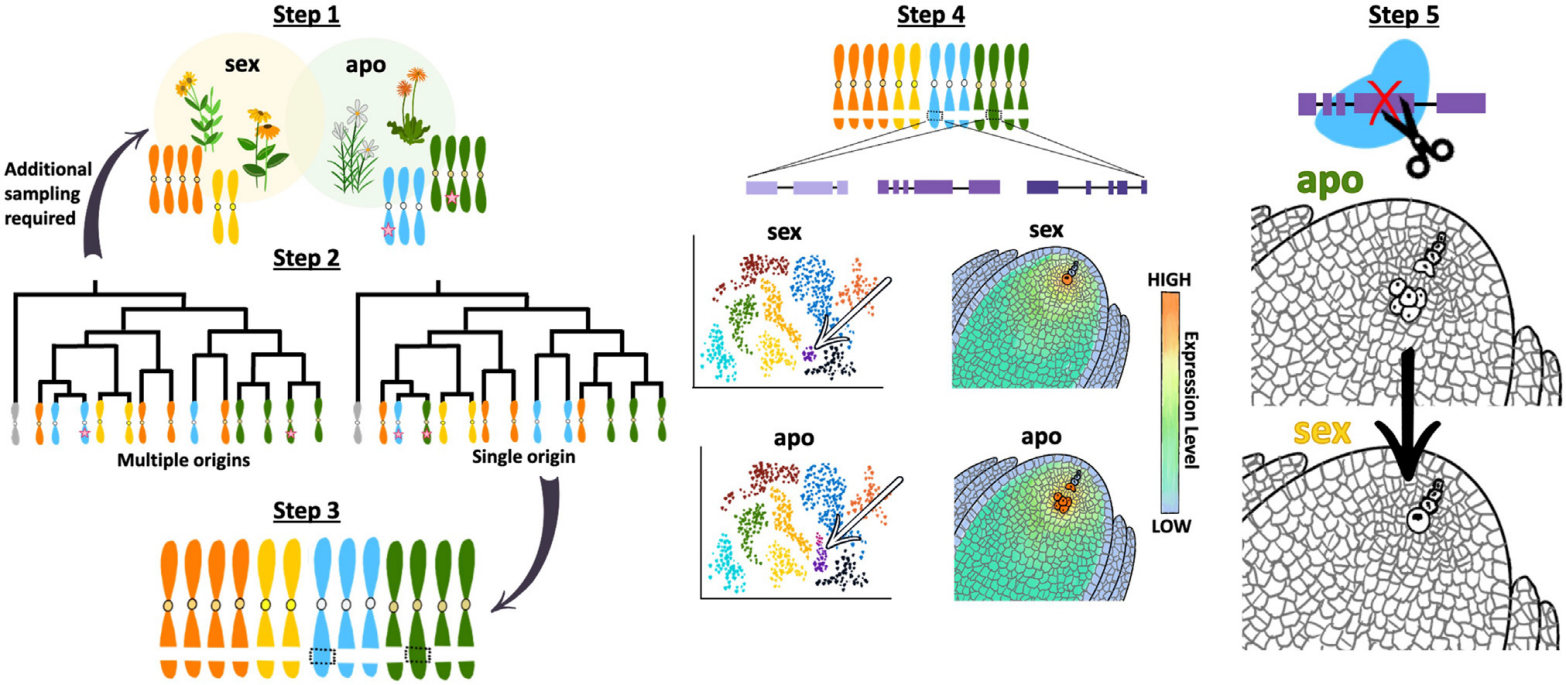
How comparative -omics and emerging technologies will accelerate the rate of apomixis gene identification Step 1: select related sexuals (sex) and apomicts (apo) for sequencing and haplotype-resolved genome assemblies. Given apomictic alleles are usually dominant, it is expected at least one haplotype from each apomict will carry the locus/loci responsible for asexual reproduction (pink stars). Step 2: assess the phylogenetic relationships of each individual haplotype and subgenome, treating them as separate entities with their own evolutionary patterns. If two related apomicts share the same origin of apomixis, at least one haplotype from each would form a monophyletic clade; this is the outcome represented in the hypothetical phylogeny on the right. In the phylogeny on the left, no haplotypes from the apomicts (colored green and blue) form a monophyletic clade, suggesting independent origins of apomixis. Since different origins may signify different causal genes, additional sampling of related apomicts and sexuals would be required for additional omics comparisons. Step 3: compare probable apomictic (based on phylogenetic patterns) and sexual haplotypes to identify genetic variation unique to apomictic haplotypes with a recent common ancestor. Step 4: use high-resolution techniques such as single-cell RNA sequencing (scRNA-seq) and spatial transcriptomics to further assist with identifying causal genes for apomixis and to understand the molecular processes changed between sexual and apomictic reproduction. scRNA-seq may identify variable cell populations in the ovule between apomicts and sexuals (white arrow); however, this technique results in a loss of spatial information. Developing and testing marker genes with traditional methods like RNA *in situ* hybridization would be necessary to confirm cells’ positions. A much more powerful method would be the implementation of spatial transcriptomics for gene candidate identification. Given the limitations of each method, a combinatorial approach could be taken. All transcriptomic data can be related back to the genetic variation to choose promising gene candidates for future functional validation. Step 5: use targeted mutagenesis techniques such as CRISPR-Cas9 to functionally validate candidate genes. In this example, a knockout of a candidate for apospory results in the transition from apomeiosis to sexual reproduction.

Easily the largest hurdle for apomixis gene discovery is the lack of genomic resources for complex, polyploid apomicts, for which no reference-quality genomes exist. However, we are now well equipped to remedy this problem with third-generation sequencing technologies and comparative genomics. PacBio HiFi and Oxford Nanopore long-read sequencing regularly enable the complete sequencing of large repetitive genomic regions, and the production of haplotype- and subgenome-resolved genome assemblies is becoming routine, even for polyploids.^82^^,^^83^^,^^84^^,^^85^^,^^86^ Targeted efforts to create assemblies of related sexuals (individuals that reproduce exclusively via sex) and apomicts followed by whole-genome sequence alignments should reveal regions of subgenomes or haplotypes that are unique to apomicts. If the regions are hemizygous, synteny comparisons of haplotypes within apomicts should provide further clues on the origin of these loci. Researchers should, however, be cautious that genomic analyses are phylogenetically informed since the convergent nature of apomixis presents the possibility of different causal genes in divergent species (Figure 3). In other words, lineage-specific information on the evolution of apomixis is needed to prevent figurative and literal comparisons, for example, of crab apples (exhibiting apospory) to oranges (exhibiting adventitious embryony).

Evidence suggests apomictic reproduction is likely caused by genes with altered spatiotemporal expression patterns residing in duplicated regions of genomes that share partial synteny with sexual species.^10^^,^^11^^,^^22^ These observations support the theory that apomixis is a deviation from sexual reproduction and that the latter represents the ancestral state.^87^ Questions on when apomixis emerges and for how long it persists in certain populations can be answered with increasing amounts of genomic resources and molecular dating techniques. Understanding the stability of apomixis in nature should better ensure its stable inheritance in crop-breeding programs. At a finer scale, it is also critical that we understand the dynamics of apomictic loci within genomes. On several occasions, these loci have been likened to the sex-determining regions (SDRs) in dioecious flowering plants, and both apomixis and dioecy exhibit convergent patterns of evolution.^88^ SDRs are also characteristically repeat rich, recombination suppressed, and sometimes hemizygous,^89^^,^^90^ and related species may show size, structural, and even location variation for sex-linked regions as sequences are accumulated and lost.^91^ While it remains largely unknown if apomictic loci show similar genome dynamics, such active processes may explain the high birth-death rate of apomixis across lineages (Figure 1). The study of these dynamics may also help describe the variation in penetrance of apomictic traits under certain environmental conditions, as the accumulation of repetitive sequence is associated with transcriptional silencing.^92^

### Spatially resolved transcriptomics to identify apomixis-related genes

Advances in transcriptomics should both (1) inform the selection of candidate genes for future functional validation within apomixis-associated loci and (2) aid in our understanding of incomplete penetrance under certain conditions. The first point has already successfully been put into practice by examining gene expression patterns around the time parthenogenesis occurred in dandelion.^10^ While bulk RNA sequencing has been useful to date, spatial transcriptomics has the potential to revolutionize the field. For example, apomeiosis usually occurs in a single cell. Observations in the cell’s spatial context at several developmental time points and gene expression comparisons between related sexual and apomictic individuals should divulge the molecular signals necessary for apomictic events to occur and assist with candidate gene selection. Similar reasoning applies to parthenogenesis and endosperm formation. Presently, most unbiased spatial transcriptomic techniques suffer from a lack of cellular resolution; however, even this limitation is lifting with technologies like scStereo-seq^93^^,^^94^ and the recently announced 10× Genomics’ Visium HD (10× Genomics, Pleasanton). However, neither the predecessor nor this new technology has been successfully applied in plant tissues. Depending on the strength of the candidate genes identified and the availability of equipment, other options for studying gene expression associated with the components of apomixis include MERFISH (multiplexed error-robust fluorescence in situ hybridization)^95^ and combinatorial approaches incorporating single-nuclei RNA sequencing and RNA *in situ* hybridization. While these techniques would significantly narrow down a list of candidate genes for apomictic traits, the last step prior to applying genes to crops would be functional validation using targeted mutagenesis. Techniques such as CRISPR-Cas9 to create null alleles would enable testing of promising candidates (Figure 3).

## Outstanding challenges for broader use of apomixis in agriculture

In summary, further study of natural apomicts is needed to increase the momentum pioneers in this field have gained so far. Although we are beginning to see promising results in a few species, widespread use of apomixis in agriculture requires that we expand the breeding tool kit through the discovery of apomictic genes in multiple lineages. To this end, we have identified major challenges that must be addressed (see Box 1). Since apomixis is a convergent evolutionary trait with more potential cases waiting to be discovered, ample opportunities exist for gene discovery. Flow cytometry seed screens have proven useful as a high-throughput way to identify natural apomicts,^96^ and low-pass sequencing methods are the next to be applied^97^ to compare progeny and mother genotypes. More genomic resources are needed for apomicts, and the selection of genotypes for genome assembly should be based on their relatedness to agriculturally important sexuals and phylogenetic patterns of the origin(s) of apomixis in these lineages. Assembling genomes of related species representing sexual reproduction and asexual reproduction, followed by comparative genomics and transcriptomics, should identify genetic variation unique to asexual individuals (Figure 3). From our perspective, directing resources toward these approaches is the most promising way to identify single genes controlling components of apomixis—including apomeiosis—and ensuring the expected functions of genes between apomicts and their engineered or introgressed sexual relatives. In other words, it is expected that these directions will result in better ease of application and reduced pleiotropic effects.

Box 1 **Future directions and challenges.**

• Generation of haplotype-phased and subgenome-resolved genomes of natural apomicts for comparative omics
• Identification of dominant apomeiosis-related genes in natural apomicts to develop a single gene-controlled dominant *MiMe* system
• Identification of more “apomixis” genes and promoters including those to engineer parthenogenesis as synthetic apomixis likely has to be adapted to every crop
• Identification of autonomous endosperm genes to facilitate the adoption of pollen-sterile apomictic plants to prevent pollen transfer and introgression into sexual crop fields
• Production of clonal seeds without decreasing fertility
• Generation of synthetic apomixis that is stable over generations and insensitive to environmental conditions
• Application of synthetic apomixis in more crops, especially in eudicots and where hybrids are difficult to generate

Finally, related to its stable integration into crop breeding programs, additional research into the incomplete penetrance of apomixis is sorely needed. Most apomicts reproduce through facultative apomixis, where both sexual and apomictic pathways occur in the same individual. On multiple occasions, researchers have shown asynchronous development and/or environmental conditions associated with the dominant mode of reproduction in facultative apomicts.^13^^,^^14^^,^^15^^,^^98^^,^^99^ This variability is a repeated feature in natural apomicts, so in order to stably integrate these genes and pathways into related crop species, highly and lowly penetrant apomictic genotypes should be prioritized for further study. One high-resolution strategy would be to first identify and functionally validate genes involved in apomictic production (using the strategies detailed in Figure 3) and then examine the transcriptional and epigenetic changes in apomicts with variable penetrance in contrasting environments, especially considering factors such as photoperiod and temperature.

The field owes its success to the immense work of several generations of scientists who intensely studied and developed model systems for apomixis. By complementing this work with additional study of natural apomicts across the angiosperm phylogeny and high-quality genomic and transcriptomic resources, we are convinced that new strategies and tools for the application of synthetic apomixis in diverse crop plants will be feasible soon.

## Acknowledgments

X.Z. was supported by a fellowship of the 10.13039/100005156Alexander von Humboldt Foundation. C.Z.G. is supported by a National Science Foundation Postdoctoral Research Fellowship in Biology, award number 2305693. A.H. is supported by a National Science Foundation Plant Genome Research Program CAREER (award number 2239530).

## Author contributions

All authors conceived, wrote, and edited the manuscript.

## Declaration of interests

The authors declare no competing interests.
